# Conformational dynamics of adenylate kinase in crystals

**DOI:** 10.1063/4.0000205

**Published:** 2024-02-21

**Authors:** Junhyung Kim, Sojin Moon, Tod D. Romo, Yifei Yang, Euiyoung Bae, George N. Phillips

**Affiliations:** 1 Department of Agricultural Biotechnology, Seoul National University, Seoul 08826, South Korea; 2 Department of Biochemistry and Biophysics, University of Rochester Medical Center, Rochester, New York 14642, USA; 3 Departments of BioSciences, Rice University, Houston, Texas 77005, USA; 4 Research Institute of Agriculture and Life Sciences, Seoul National University, Seoul 08826, South Korea; 5 Department of Chemistry, Rice University, Houston, Texas 77005, USA

## Abstract

Adenylate kinase is a ubiquitous enzyme in living systems and undergoes dramatic conformational changes during its catalytic cycle. For these reasons, it is widely studied by genetic, biochemical, and biophysical methods, both experimental and theoretical. We have determined the basic crystal structures of three differently liganded states of adenylate kinase from *Methanotorrus igneus*, a hyperthermophilic organism whose adenylate kinase is a homotrimeric oligomer. The multiple copies of each protomer in the asymmetric unit of the crystal provide a unique opportunity to study the variation in the structure and were further analyzed using advanced crystallographic refinement methods and analysis tools to reveal conformational heterogeneity and, thus, implied dynamic behaviors in the catalytic cycle.

## INTRODUCTION

The relationship between the internal movements of enzymes and their catalytic activities is complicated. Stochastic complexity abounds, leading to energy landscapes with fluctuations in molecular confirmations that have states that can either promote or inhibit the binding of substrates, enable actual chemical catalysis, or cause the unbinding of products. While we have strong methods for determining average structures of enzymes and key intermediates, methods for identifying motions and their relevance at the individual molecule level are limited. One widely studied model system for structure–dynamics–function relationships is the small protein, adenylate kinase (AK), a ubiquitous, small enzyme that catalyzes the transfer of a phosphate group between adenosine nucleotides to help us maintain levels of ATP in cells (Barkulis and Lehninger, 1951),

ATP + AMP↔2 ADP.

It has a common architecture across all life forms, based on sequence and structural homologies, comprising a relatively static core and dynamic loops of various sizes that can close around the site of phosphoryl transfer (Schlauderer *et al.*, 1996). Archaeal adenylate kinases exhibit trimeric assembly (Criswell *et al.*, 2003), whereas bacterial and eukaryotic homologues are monomeric (Bae and Phillips, 2004; Moon *et al.*, 2017).

**FIG. 1.**
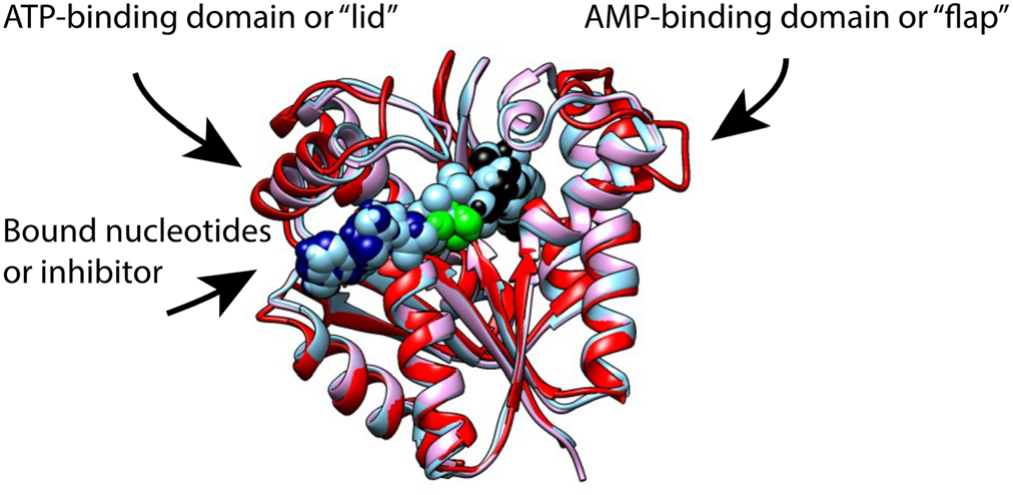
Conformational changes of *M. igneus* AK in different liganded states superposed. On average, the lid and flap move in to close around the substrate/inhibitor. The apo form of the enzyme is shown in red, the Ap_5_A-bound form in pink, and the AMP-bound form in cyan.

The reversible reaction coordinate of adenylate kinase catalysis includes binding of an AMP to a site we refer to as the AMP-binding domain or “flap,” and binding of an ATP-Mg++ moiety to another site that comprises a “lid” domain that can also be closed on a bound substrate (Fig. 1). Early kinetic studies on the rabbit muscle and on the yeast adenylate kinase suggested a random bi–bi enzymatic mechanism, at least for the enzyme from these species, so that either substrate can bind first (Rhoads and Lowenstein, 1968; Schulz *et al.*, 1974; and Khoo and Russell, 1970). The departure of the pair of nucleotides and the magnesium ion appear to be the rate-limiting steps in the overall reaction and from various crystal structures these products are sterically restricted from leaving unless the flap and the lid at least partially open or unfold (Berry *et al.*, 2006; 1994; and Berry and Phillips, 1998).

The detailed changes in structure that lead to the phosphoryl transfer includes a critical role for the guanidinium group of an arginine side chain [numbered 132 in the chicken sequence (Dahnke *et al.*, 1992) but numbered 138 in our structures] and whose position is important in stabilizing an anionic phosphorane or metaphosphate intermediate (Allen and Dunaway-Mariano, 2004). Furthermore, it has also been recently postulated that a cation–pi interaction between another arginine side chain (numbered 119 in *Escherichia coli* and 131 in *Methanotorrus igneus*) and the adenine ring at the ATP site plays a role in the closing of the lid domain in the *E. coli* form of AK. (Rogne *et al.*, 2019). The large scale motions and mechanistic theory described here have been supported, and more detailed hypotheses are generated by molecular dynamics simulations, NMR, thermodynamic studies, mutagenesis, and many others (Daily *et al.*, 2010; 2013; Kerns *et al.*, 2015; Schrank *et al.*, 2013; and Whitford *et al.*, 2007).

The stochastic complexity of this system suggests that one approach to obtaining a better understanding of the mechanism would be to define the energy landscapes of the different states and to identify likely pathways for a successful catalytic event. Simulations have been developed along these lines based on contact map based potentials (Daily *et al.*, 2013; Zheng and Cui, 2018) and seem to show a distributed set of transitions rather than a simple hinge-bending mode, supporting more a local unfolding mechanism over a single defined pathway.

The overall average structural transitions that occur when various substrates or inhibitors bind to adenylate kinase are well determined and described. There are over 130 Protein Data Bank (Berman *et al.*, 2000) depositions with EC 2.7.4.3 (Enzyme Commission number for adenylate kinase) in the description, representing more than 40 bacterial, eukaryotic, and archaeal species. Biochemical studies show that AMP and ADP can bind in the ATP site, resulting in substrate inhibition (Adkar *et al.*, 2011) and prior structures from some species exist in this form (Berry *et al.*, 2006; Criswell *et al.*, 2003; Kerns *et al.*, 2015; and Vonrhein *et al.*, 1998). A commonly employed inhibitor often added during crystallization is P^1^,P^5^-di(adenosine 5′)-pentaphosphate (Ap_5_A). Bone *et al.* (1986) comprising two adenylyl groups with a five-phosphate connecting chain instead of the transition state-like four phosphate moieties. It locks the enzyme in a folded, mostly closed conformation. Ap_5_A cannot be hydrolyzed presumably because of the additional phosphate group that distorts the active site. Most crystal structures of adenylate kinase are closed forms with Ap_5_A bound, likely because the structure is more rigid and, thus, more likely to crystallize, as demonstrated by the plethora of AK structures that have been determined. Crystal structures of adenylate kinase in its apo form are relatively more rare (Buchko *et al.*, 2010; Kovermann *et al.*, 2015) but seem to be more common for AK from thermophilic species, which also tend to have a relatively more stable trimeric arrangement of core domains (Bae and Phillips, 2006). Static crystal structures of various intermediate states are also known for some species, including the nontransferable substrate AMPPNP (Berry *et al.*, 1994). Perhaps the most interesting structures from the standpoint of transition pathways are those where intermediates of the open-closed transition happen to be trapped by the crystallization process (Kerns *et al.*, 2015).

We present here an experimental crystal structure analysis of three differently bound states of adenylate kinase (AKign) from the same species, *M. igneus,* formerly named *Methanococcus igneus*. Multiple copies of the protein exist in the asymmetric unit of the crystal and ensemble refinements allow us to map aspects of the energy landscape of the enzyme in these crystals. The presence of many copies in the asymmetric unit allows the examination of a number of states in different packing environments of the crystal. Ensemble refinements of the structures (refs) allow an alternative visualization of the variability in the more mobile parts of the structure, and in theory, represent a more realistic analysis of the crystallographic variability than simply describing the amplitude of variability with Gaussian-shaped B-factors. These refinements often represent a trade-off between the physical accuracy of the individual structures and the fit to the diffraction data, but the R_free_ values suggest that they are valid descriptions of the structure and its variations (Levin *et al.*, 2007).

## MATERIALS AND METHODS

### Cloning, expression, and purification

The synthetic gene of AKign was cloned into a pET21a vector with a C-terminal (His)_6_ tag. *Escherichia coli* BL21 (DE3) cells transformed with the AKign construct were cultured in LB medium at 37 °C until the optical density reached 0.7 at 600 nm. Protein expression was induced by the addition of 0.5 mM isopropyl-β-D-thiogalactopyranoside for 6 h. The cells were harvested by centrifugation and resuspended in the purification buffer (500 mM NaCl, 3 mM β-mercaptoethanol (BME), 10% (w/v) glycerol, and 20 mM Tris-HCl pH 7.0).

After sonication and centrifugation, the supernatant was incubated at 65 °C for 30 min, and denatured *E. coli* proteins were removed by centrifugation. The sample was loaded onto 5 ml HisTrap HP column (GE Healthcare) pre-equilibrated with the purification buffer. After washing the column with the buffer, the bound protein was eluted by applying a linear gradient of imidazole (up to 500 mM). The AKign protein was further purified using a HiLoad 16/60 Superdex75 column (GE Healthcare) equilibrated with the size-exclusion chromatography buffer [100 mM NaCl, 3 mM dithiothreitol (DTT), 5% (w/v) glycerol, and 50 mM HEPES pH 7.0].

### Crystallization

All AKign crystals used in this study were grown at 20 °C using the sitting-drop vapor diffusion method from 20 mg/ml protein mixed with an equal volume of reservoir solution. Crystals of AKign without bound ligands were obtained with the reservoir solution containing 30% (v/v) MPD and 100 mM Tris-HCl pH 8.0. Crystals of AKign bound to two AMP molecules were grown at the condition containing 4 mM AMP, 8% (w/v) polyethylene glycol 8000, 100 mM magnesium acetate, and 100 mM sodium acetate pH 4.5. Crystals of Ap_5_A-bound AKign were obtained by using the reservoir solution including 4 mM Ap_5_A, 17% (w/v) polyethylene glycol 3350, and 250 mM sodium malonate. The crystals were either flash-frozen in liquid nitrogen without any cryoprotecting reagents (for apo and AMP-bound structures) or cryoprotected in the reservoir solutions supplemented with 30% ethylene glycol (for an Ap_5_A-bound structure).

### Data collection and standard structure determination

Diffraction data were collected at the beamline 7A of the Pohang Accelerator Laboratory at 100 K. Data were processed with XDS (Kabsch, 2010), molecular replacement was performed using PHENIX (Adams *et al.*, 2011) starting with the AK structures from *M. voltae* and *M. thermolithotrophicus*. All three structures were built and refined with Coot (Emsley and Cowtan, 2004) and PHENIX. The entire sequence was built for all three crystal forms from the native N-terminal residue to the native C-terminal lysine with the disordered C-terminal his tag left unbuilt. For the apo form, the lid region was particularly difficult to fit with a single structure and several residues were omitted from the apo version of the PDB depositions. The AMP and Ap5A structures were left complete despite occasional poor real space real space R-values in order to proceed with the ensemble refinements (Table I). For the ensemble refinements, a version of the apo-form with complete lid domains was used (see below). To prepare for the ensemble refinements, TLS was used with one chain per group and group ADPs with two parameters per residue.

**TABLE I.** Data collection and refinement statistics.^a^

	Apo	AMP	Ap_5_A
No. of trimers in asymmetric unit	2	4	2
No. of ligands per protomer	0	2	1
Space group	P3_1_	P2_1_	P2_1_
Unit cell parameters (Å)	a = 68.7, b = 68.7, c = 258.0	a = 88.8, b = 121.4, c = 121.0, β = 103.0°	a = 67.5, b = 122.5, c = 83.6, β = 100.2°
Wavelength (Å)	0.9793	0.9793	0.9793
**Data collection statistics**			
Resolution range (Å)	48.9 – 2.30 (2.44–2.30)	44.3 – 2.40 (2.46–2.40)	33.2 – 2.25 (2.39–2.25)
Number of reflections	60 464 (9674)	92 368 (2016)	59 223 (1976)
Completeness (%)	99.9 (98.9)	94.5 (72.0)	93.4 (70.8)
CC_1/2_^b^	99.8 (40.2)	99.6 (24.4)	99.98 (28.2)
Redundancy	5.89 (5.34)	7.11 (5.28)	3.85 (2.75)
Mean I/σ	9.08 (1.04)	10.51 (0.77)	10.898 (0.57)
**Refinement statistics**			
Resolution range (Å)	31.33 – 2.30	44.33 – 2.40	33.19 – 2.25
R_cryst_^c^/R_free_^d^	0.229/0.267	0.200/0.248	0.215/0.256
RMSD bonds (Å)	0.02	0.02	0.02
RMSD angles (deg)	1.54	1.57	1.75
Average B factor (Å^2^)	70.0	66.81	80.0
Number of water molecules	139	164	43
Ramachandran favored (%)	96.95	98.32	95.68
Ramachandran allowed (%)	2.87	1.59	4.06
RSCC (ligands)^e^	n/a	0.86–0.99	0.91–0.95

^a^ Values in parentheses are for the highest-resolution shell.

^b^ CC_1/2_ = percentage of correlation between intensities from random half-datasets.

^c^ R_cryst_ = Σ_h_ǁF_obs_| – |F_calc_ǁ/Σ_h_|F_obs_|, where F_obs_ and F_calc_ are the observed and calculated structure factor amplitudes, respectively.

^d^ R_free_ was calculated as R_cryst_ using 2%–4% randomly selected unique reflections omitted from structure refinement.

^e^ RSCC is the range of real space correlation coefficients for the nucleotide ligands.

### Ensemble refinement

The PHENIX suite of programs (Adams *et al.*, 2010) was used to perform the ensemble refinements. The initial refinements were carried out by using a complete trace of the protein (no missing loops). Once these structures had converged, including placement of explicit solvent molecules, the ensemble protocol was invoked. As suggested in the Phenix documentation, a range of values for the variables pTLS (fraction of the structure used to set the TLS regions, tx (integration time for the molecular dynamics, and temperature offset (relative weight of the x-ray data) were examined. For all three cases, the best parameters were found to be tx = 2, offset = 5, and pTLS =0.75. Analysis of the geometry of the members of the default reduced set of ensemble structures is given in Table S3. For the generation of the large ensembles for SVD analysis, more blocks were used (50 instead of the default of 1) and the usual reduction in the ensemble to a small set of representative structures was not performed (Table S3).

### Variation analysis

The root mean square deviations from the average of the various coordinate sets were performed using the ProFit program (Martin and Porter, unpublished), using pdb-tools (http://www.bioinf.org.uk/software/profit) to extract coordinates of different subunits across the many models in the ensemble refinements. Excel (Microsoft) was used to display the pairwise matrix, and the default graded three-color (green–white–red) scale conditional color formatting was used.

## RESULTS

### Crystal structures of AKign in various liganded states

To study structural dynamics of AKign during catalysis, we determined crystal structures of AKign in three different liganded states (Fig. 1). Data collection and refinement statistics are summarized in Table I. First, we crystallized AKign in the absence of any ligands, which led the determination of the unliganded, apo structure. By adding 4 mM AMP in the crystallization buffer, we were able to obtain the AMP-bound AKign structure, which comprises two bound AMP molecules per AKign protomer. We also solved the AKign structure with the bound inhibitor, Ap_5_A. The structure determinations revealed all of the AKign forms to comprise trimers, in which three protomers are arranged with strong but not exact threefold symmetry. Furthermore, in the asymmetric units of the crystals, the apo and Ap_5_A-bound structures comprise two AKign trimers, whereas the AMP-bound structures comprise four trimers, thus allowing an unusually broad comparison of the variability in conformations to be explored.

**FIG. 2.**
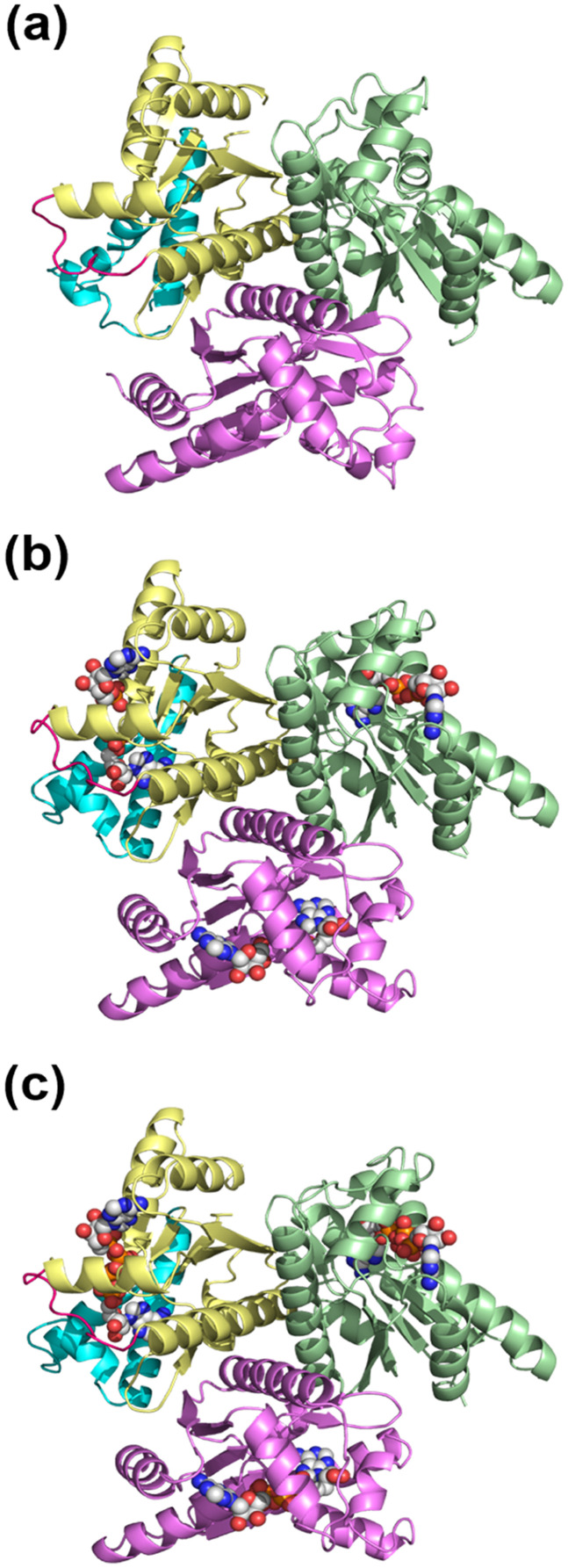
Trimeric structures of AKign. Crystal structures of apo (a), AMP-bound (b), and Ap_5_A-bound forms. (c) The trimers are viewed in the same orientations. In each of the top-left protomeric subunits, the CORE (residues 1–38, 86–134, and 145–192), AMP_bind_ (residues 39–85), and LID (residues 135–144) domains are shown in yellow, cyan, and magenta, respectively. The other two AKign protomers in each trimer are shown in green and pink, respectively. The bound ligands are represented in the space-filling models (C, gray; N, blue; O, red; and P, orange). The result show that the overall trimeric arrangement is not altered in crystal forms in various liganded states.

The chain folds of the AKign protomers in the structures are essentially identical to those of other archaeal AK structures (Fig. 1). The structure determinations reveal the characteristic three-domain arrangements within a protomer of AK: the CORE (residues 1–38, 86–134, and 145–192), AMP_bind_ (residues 39–85), and LID (residues 135–144) domains (Fig. 2). The CORE domain contains a central five-stranded parallel β-sheet (β1–3, β6, and β7) and five α-helices (α1 and α5–8). The two additional β-strands (β4 and β5) in the CORE domain extend the central β-sheet of another subunit upon multimerization, which is unique to archaeal AKs. In addition to the extension of the central β-sheets, the trimerization of AKign involves contacts between the α7 helices of each subunit, which forms a three-helix bundle stabilized mostly by hydrophobic interactions around the threefold symmetry axis (Fig. 2).

**FIG. 3.**
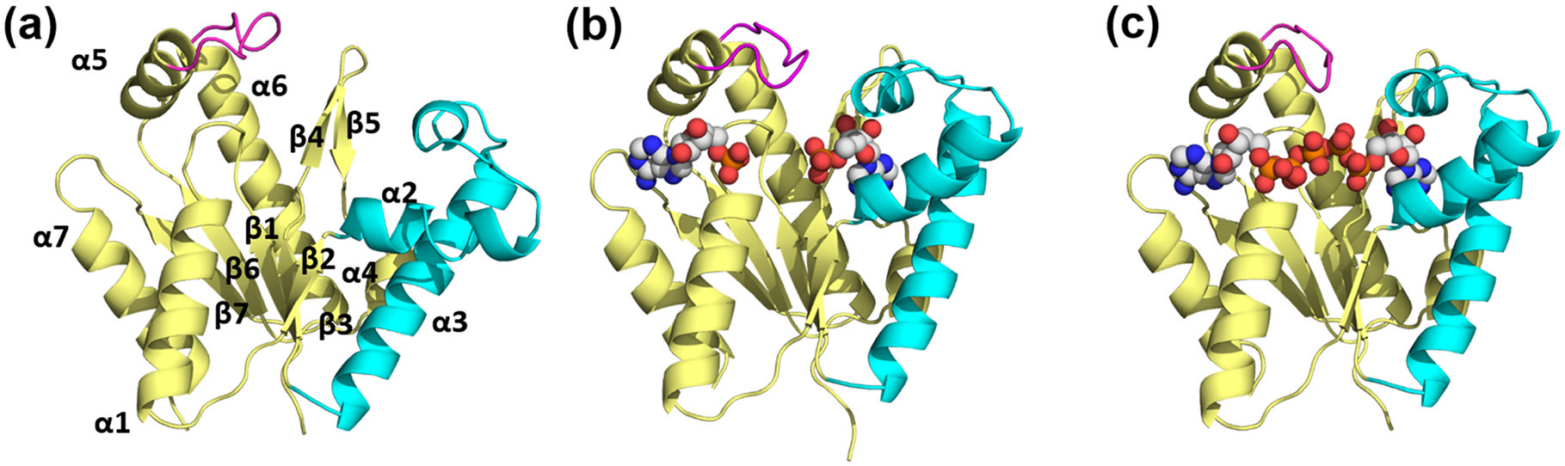
Average crystal structures of protomers of AKign. Protomeric structures of the A subunits of apo (a), AMP-bound (b), and Ap_5_A-bound AKign (c) are viewed in the same orientation. The CORE (residues 1–38, 86–134, and 145–192), AMP_bind_ (residues 39–85), and LID (residues 135–144) domains are shown in yellow, cyan, and magenta, respectively. The bound ligands are represented in the space-filling models (C, gray; N, blue; O, red; and P, orange). The AMP-bound AKign contains two AMP molecules per one AKign protomer. Secondary structural elements are indicated in the apo structure. Trimeric structures of apo, AMP-bound, and Ap_5_A-bound AKign are shown in Fig. S1.

The AMP_bind_ domain includes three α-helices (α2–4), and the LID domain is a short loop connecting α6 and α7 helices, as is true of other archaeal AK structures. In the AMP- and Ap_5_A-bound AKign structures, AMP- and ATP-binding sites are occupied by two AMPs and a single Ap_5_A, respectively, and largely covered by the AMP_bind_ and LID domains, indicating a closed conformation of AK. However, the details around the active site are different (Fig. 3).

On the other hand, the apo structure adopts a less ordered and more open conformational state. The disposition of the essential, conserved guanidinium group of Arg140 varies among the three states, both in average position and state of order. In the AMP-bound structure, it in near the water-Mg^2+^ cluster but is not well ordered and varies in position among the 12 copies in the asymmetric unit (see also below).

In the apo structure, the lid domain is quite disordered, and it was not possible to refine this region well enough to resolve an average position for Arg140. In the Ap_5_A structure, Arg140 is much more ordered (Fig. 9) and points toward the phosphate oxygens of the gamma phosphate group on the ATP side, the one that is transferred to AMP during the phosphotransferase reaction. As described by Rogne *et al.* (2019), our liganded structure also has an arginine positioned for a cation–pi interaction with the adenine in the ATP; however, our apo structure reveals the guanidinium group to be more or less in the same position as when nucleotides are bound but is somewhat less ordered. We also see a potential interaction between the sulfur atom of Met43 and the N3 atom of the adenine ring on the AMP side, which may form a similar substrate binding role on the other side of the active site.

### Ensemble refinement of AKign

Adenylate kinases have been the subject of many computational and experimental studies of protein dynamics (Elamrani *et al.*, 1996; Halder *et al.*, 2017; Kern *et al.*, 1994; Krishnamurthy *et al.*, 2005; Krishnamurthy *et al.*, 2009; Kubitzki and de Groot, 2008; Li *et al.*, 2015; Lou and Cukier, 2006; Lu and Wang, 2008; Ono *et al.*, 2015; Ping *et al.*, 2013; Pontiggia *et al.*, 2008; Sanders *et al.*, 1989; Shapiro *et al.*, 2000; Snow *et al.*, 2007; Song and Zhu, 2013; Temiz *et al.*, 2004; and Unan *et al.*, 2015), but the range and the roles of its motions are still incomplete and or controversial. To further examine the range of conformations that occur around the active site during catalysis, we performed ensemble refinements (Burnley *et al.*, 2012; Forneris *et al.*, 2014; Levin *et al.*, 2007; and Schiffer *et al.*, 1995) using the measured diffraction amplitudes and protocols in the Phenix refinement package (Adams *et al.*, 2011). These refinements represent a visualization of the range of structures as an alternative providing a single model with large B-factors or unmodeled sections of the protein. The process involves using a molecular dynamics trajectory to expand the atomic model in areas where the electron density is weak to include a set of structures that, taken together, provide a somewhat better description of the structure than a single static model with assumed Gaussian deviations in atomic displacements. One can display the actual structures resulting from the ensemble refinements as a series. Although the time component may be lost, the range of structures can be examined for interesting features.

**FIG. 4.**
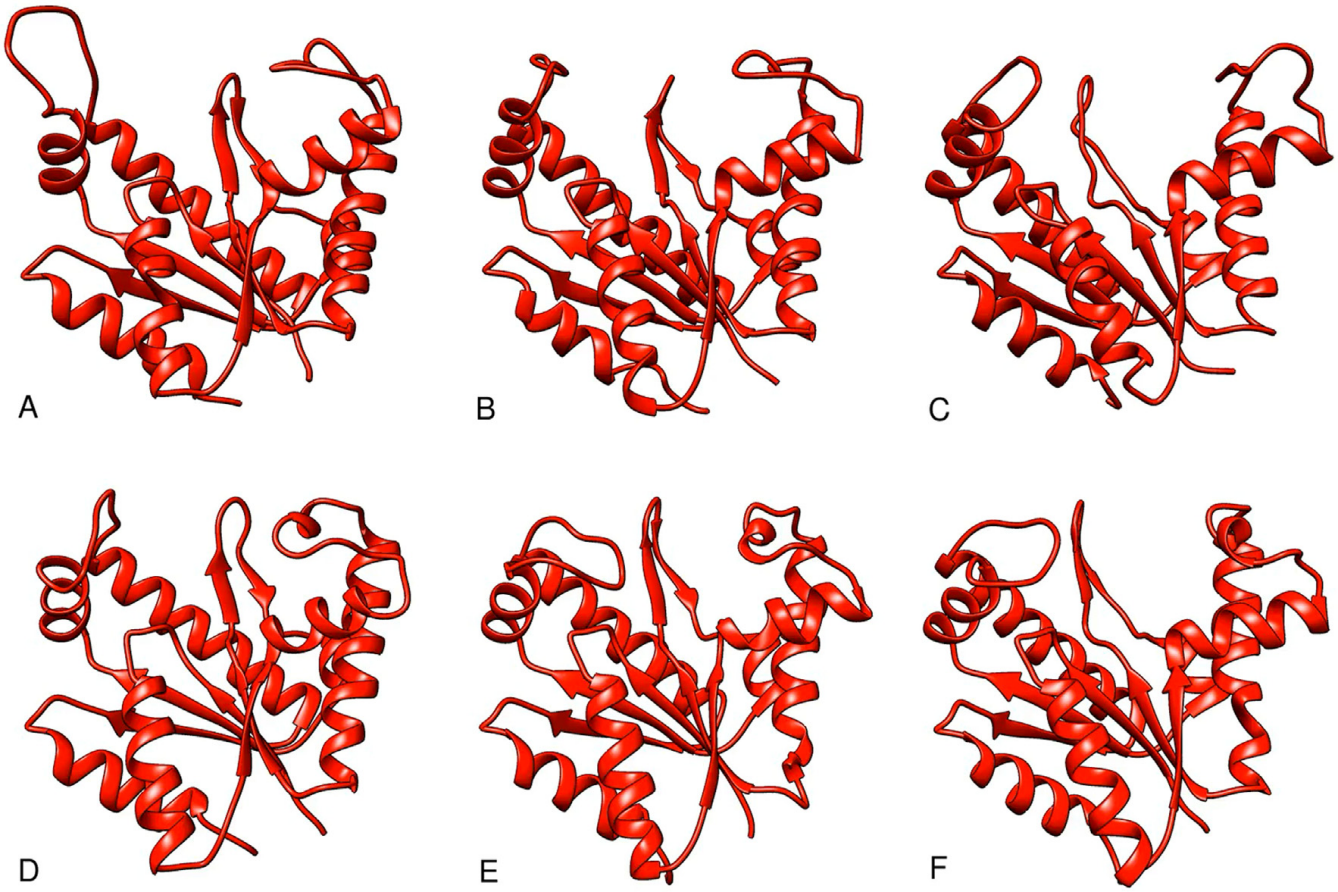
Video-based ensemble refinement results for the apo state of AKign. Both the lid domain and the AMP-binding flap domains are quite open and show a dramatic range of conformations. A static image is available in the supplementary material. The structures are arranged by chain Id with A,B,C and D,E,F, each comprising the subunits of a trimer, but separated into protomers for easier comparison. Multimedia available online.
10.1063/4.0000205.1

**FIG. 5.**
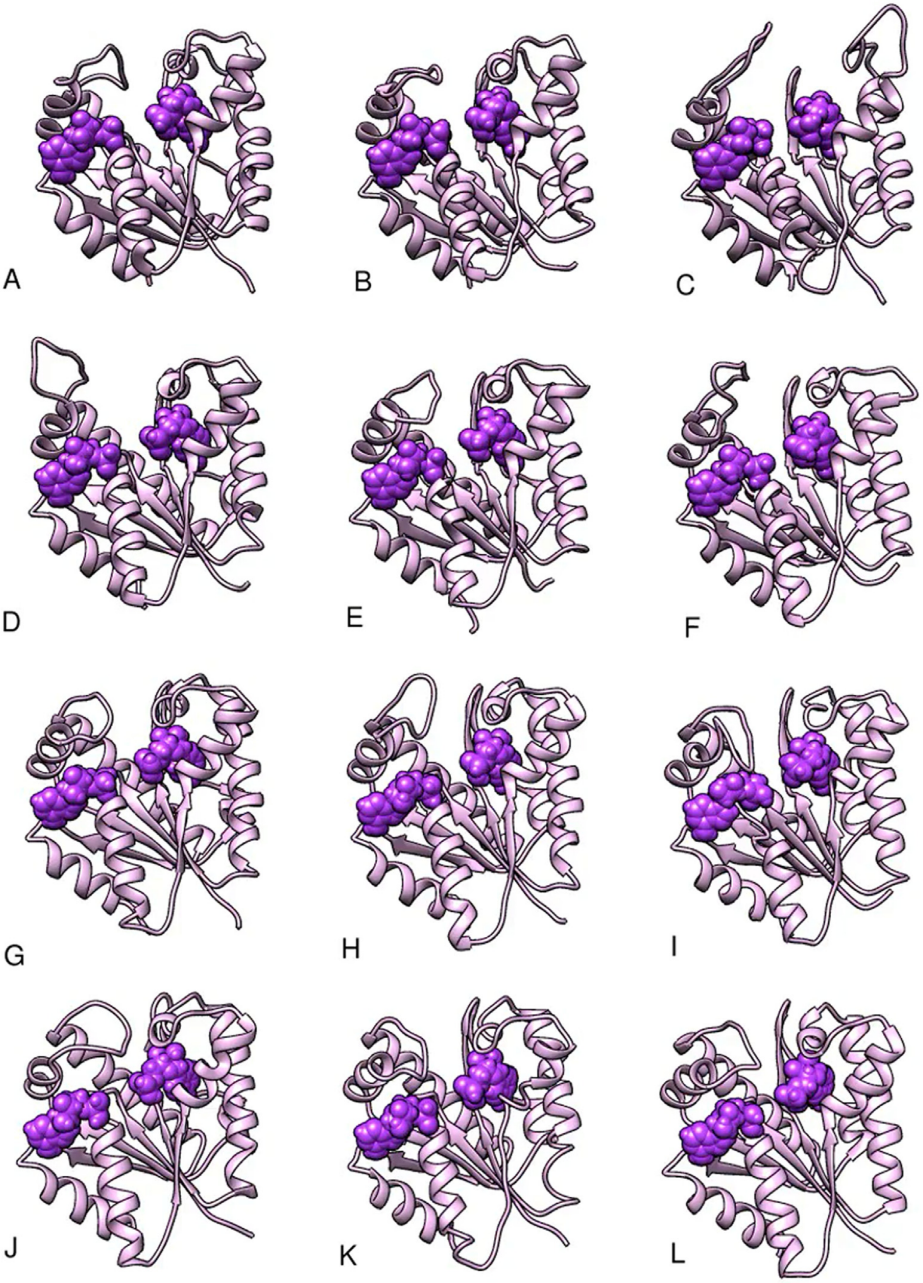
Video-based ensemble refinement results for the AMP-bound state of AKign. The two molecules of AMP are shown in purple. The AMP-binding flap domains show a smaller range of conformations than the apo-form. A static image is available in the supplementary material. The structures are arranged by chain Id with A,B,C; D,E,F; G,H,I; and J,K,L each comprising the subunits of a trimer but separated into protomers for easier comparison. Multimedia available online.
10.1063/4.0000205.2

**FIG. 6.**
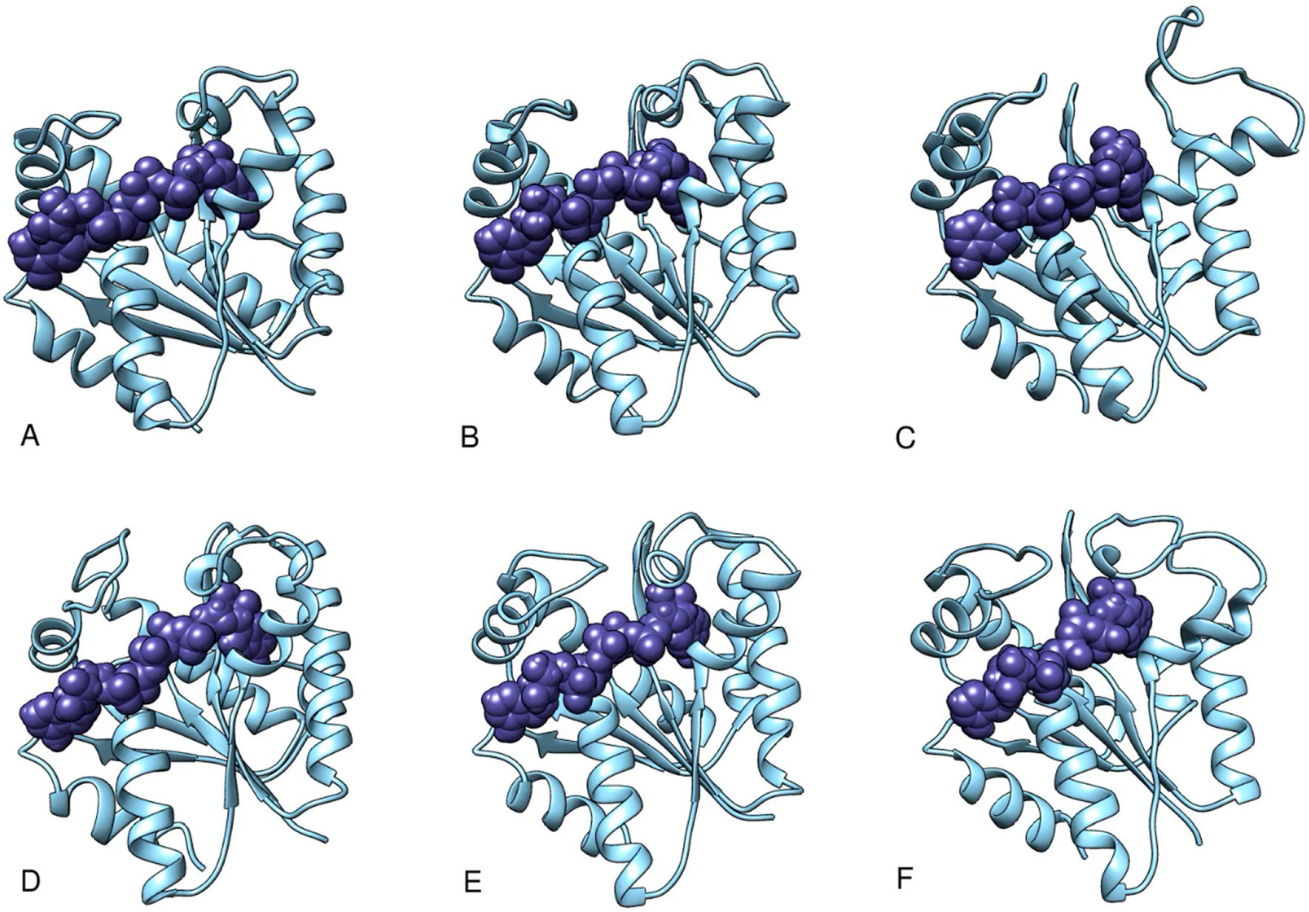
Video-based ensemble refinement results for the Ap_5_A state of AKign. The Ap5A inhibitor molecules show space-filling spheres in deep purple. Both lid and the AMP-binding flap domains show a smaller range of conformations than the other forms. A static image is available in the supplementary material. The structures are arranged by chain Id with A,B,C and D,E,F; each comprising the subunits of a trimer but separated into protomers for easier comparison. Multimedia available online.
10.1063/4.0000205.3

Our ensemble refinement results are presented for each of the crystal structures we determined. The ensemble refinements can be compared statistically with classical refinements (Table II). In each case, R_cryst_ is dramatically lower, as expected because of the increased number of effective parameters, but R_free_ also drops slightly, indicating that the ensemble representation may actually be slightly better than the single molecule version (Gros, Brunger, Levin, and Adams) or at least an equally valid way of presenting a structural analysis. The various structural ensembles are shown in Figs. 4–6 (Multimedia views). Please note that even though these images are shown as a series, no time order is to be inferred. The videos simply make it easier to visualize the differences than the superposition of a large number of structures. That having been said, it is highly likely that there are transitions between members of this ensemble at non-cryogenic temperatures, so that the general impression of mobility between the states is given.

**TABLE II.** Ensemble refinement statistics.

	Apo	AMP	Ap_5_A
TLS—single model			
R_cryst_^a^^,^^b^	0.229	0.202	0.215
R_free_^a^^,^^c^	0.267	0.248	0.256
TLS—reduced ensemble			
R_cryst_	0.200	0.163	0.177
R_free_	0.245	0.237	0.238
No. of models	16	30	16
TLS—trajectory			
No. of models	2667	3000	2667
R_cryst_	0.197	0.158	0.173
R_free_	0.246	0.231	0.233

^a^ Values in parentheses are for the highest-resolution shell.

^b^ R_cryst_ = Σ_h_ǁF_obs_|−|F_calc_ǁ/Σ_h_|F_obs_|, where F_obs_ and F_calc_ are the observed and calculated structure factor amplitudes, respectively.

^c^ R_free_ was calculated as R_cryst_ using 2%–4% randomly selected unique reflections omitted from the structure refinement.

One can see in these representations the range of motions in the lid and AMP-binding domains as a function of what is bound in the active site of the protein. Despite the differences in crystal forms, the general trend is similar. Those structures with no nucleotide bound in the active site (apo forms) show the largest range of structures and those with the inhibitor Ap_5_A bound show a somewhat narrower range around a more closed conformation. There is some variation, however, from one subunit to another within one asymmetric unit of the crystal. The multiple instances of the structural variation in slightly different environments provides an opportunity to observe the limited but real effects of crystal packing on the conformational variations in the protein structure (Kondrashov *et al.*, 2008).

### Variations within the asymmetric unit

**FIG. 7.**
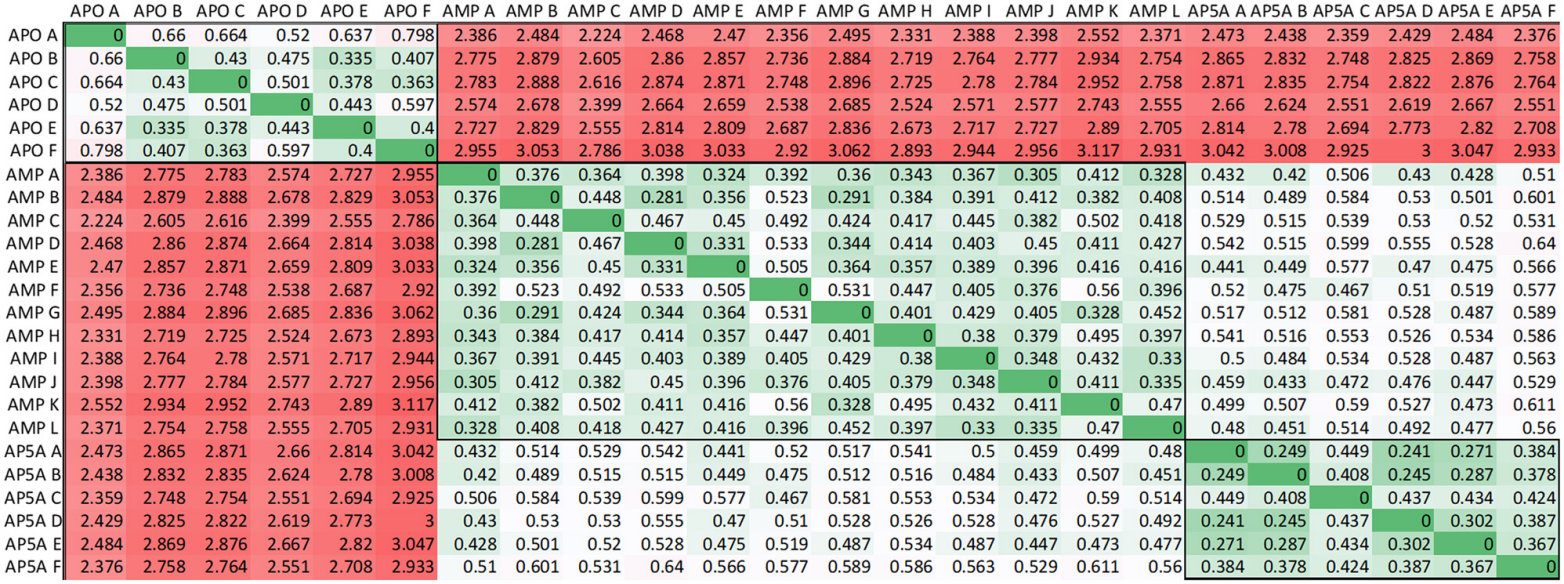
Pairwise aligned root mean square deviations of the average atomic coordinates of the various members in the asymmetric unit of the three liganded forms of AKign. The largest deviations (red) are between each copy of the apo-form and the two liganded forms (AMP and Ap_5_A). The smallest deviations (green) are for the multiple copies in the asymmetric units for each liganded form (large greenish blocks near the diagonal). The results show that the members of each liganded form are more similar to each other than to the other liganded forms. However, there is some conformation overlap between the forms with AMP and the form with Ap_5_A, shown by the existence of some small values off the diagonal. Zeros along the diagonal denote the self-comparison within one liganded form.

Since there are either six or twelve copies of the protomer in the asymmetric unit of the crystal, we can measure the variability of the structures within each liganded form and across the various liganded forms. Figure 7 shows the pairwise root mean square deviations of the atomic positions of the proteins. Clearly, the deviations within each liganded form are smaller than the differences between the three different liganded forms as blocks of similar numbers can be seen in the graph. However, the analysis also shows that while it is unlikely that protomers in the apo crystal form will have conformations similar to the bound forms, it is possible that conformations of protomers in the AMP form can mimic conformations in the Ap_5_A form and vice versa (green numbers outside the blocks in the figure). A further analysis of the space of the conformational variation instead of just the magnitude is given in the supplementary material, Fig. S3, and the related text.

### Role of arginine in the catalytic cycle

**FIG. 8.**
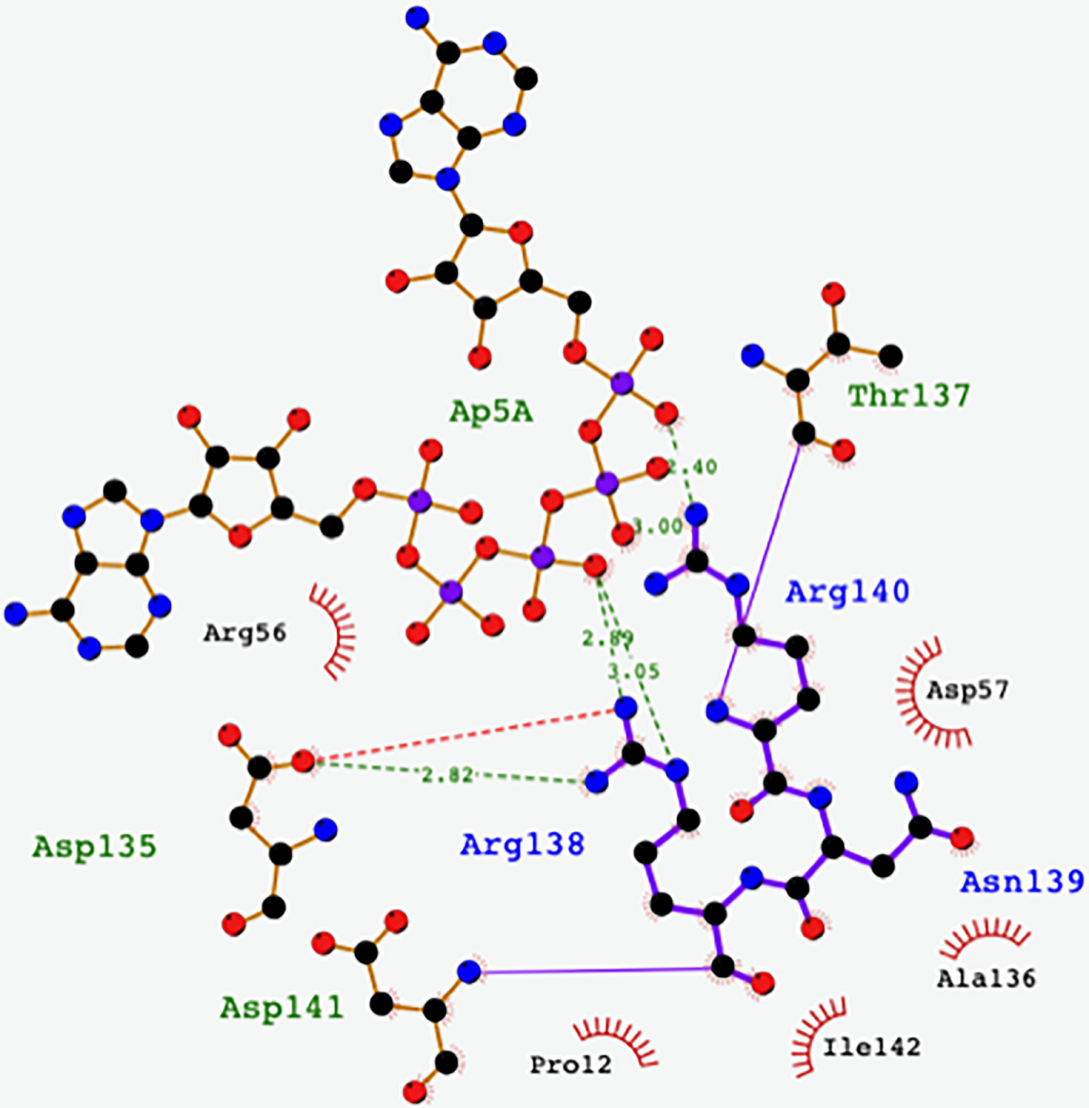
A schematic diagram of the relationship of the critical arginine side chains (blue) to the phosphate moieties and other nearby residues for the A chain of Akign. Chains A–E give a similar pattern but chain F shows the guanidinium groups farther from the phosphates. The plot is made with LigPlot+ (Wallace *et al.*, 1995).

**FIG. 9.**
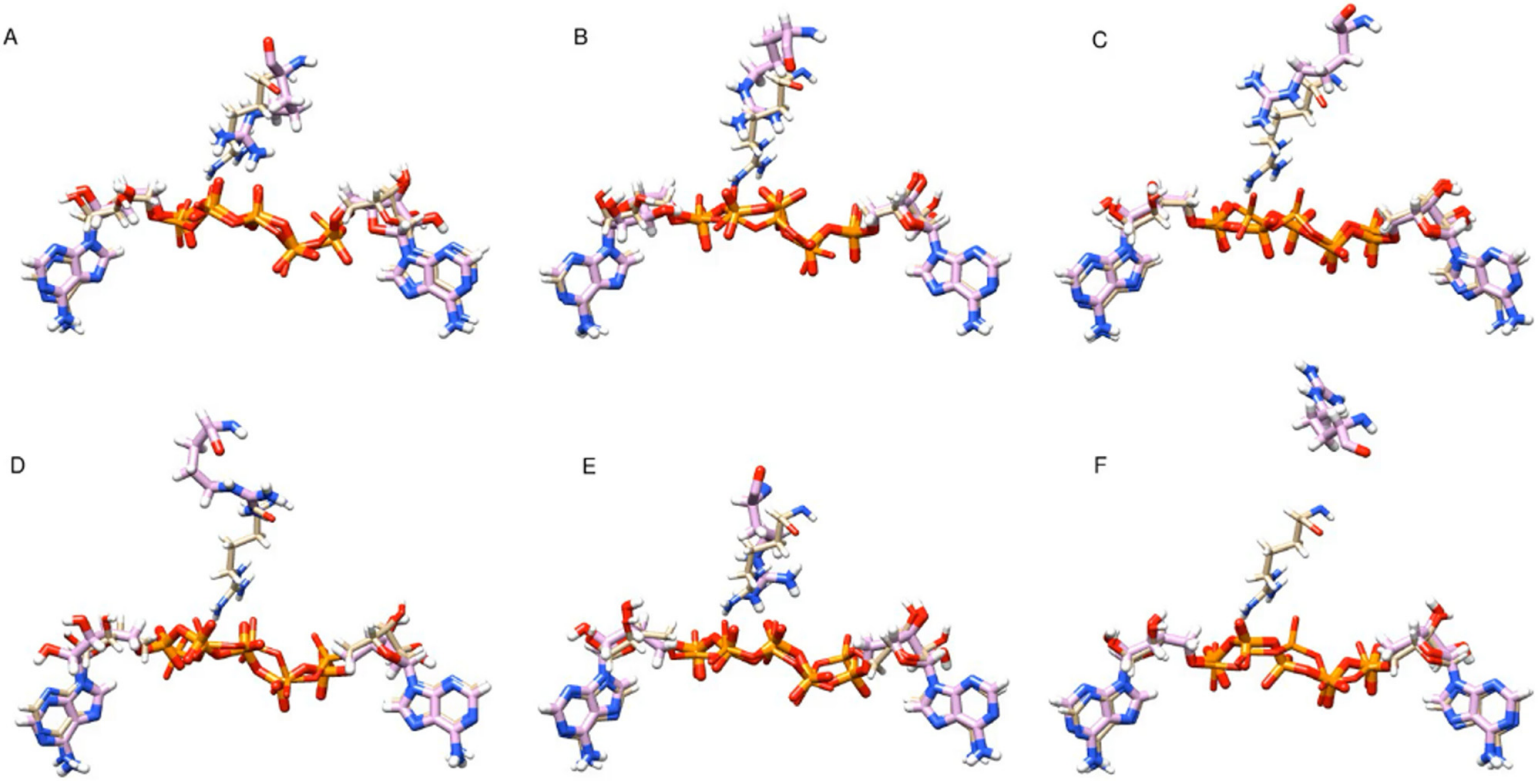
The Ap5A-bound form with the critical arginine side chain is shown. This video shows the variation in ensemble refinement locations of R138, which bounces between the oxygen atoms of various phosphate atoms in the inhibitor Ap5A. The six views of subunits A–F are shown in the movie with the various conformations from the ensemble refinements shown as a series. Multimedia available online.
10.1063/4.0000205.4

The dynamic behavior of a critical arginine side chain (R138 in our numbering system and R145 in the work cited below) supports a hypothesis describing its critical role in catalysis (Shibanuma *et al.*, 2020). Using quantum mechanics and molecular dynamics calculations, it is proposed that the phosphotransferase reaction has two energy barriers along the minimum free energy path of the phosphoryl transfer, essentially the breaking of one oxygen phosphate bond and the making of its new one. The guanidinium group of R138(145) is proposed to break and re-form hydrogen bonds with the transferred phosphate moiety. In five of the six instances in the asymmetric unit of our enzyme, the R138 side chains of AKign are seen “dancing” around the phosphates in a manner similar to the proposed mechanism. A schematic diagram showing hydrogen bonds between R138(145) and R140(147) and the Ap5A inhibitor for the A chain is given for orientation (Fig. 8) and a video is given showing the variation in position seen in the ensemble refinements [Fig. 9 (Multimedia view)].

## DISCUSSION

In general, the connections between structure, dynamics, and enzyme catalysis remain poorly understood. Adenylate kinase, despite the publication of about 1500 papers on structure, function, or dynamics, is no exception. We can design proteins to bind ligands, but we cannot yet design an enzyme. For adenylate kinase enzymology (Rhoads and Lowenstein, 1968; Khoo and Russell, 1970), genetics (Ferber *et al.*, 1997), crystallography (Berry *et al.*, 2006; Criswell *et al.*, 2003; Henzler-Wildman *et al.*, 2007; and Schulz *et al.*, 1974), solution scattering (Daily *et al.*, 2013), NMR (Kerns *et al.*, 2015) (Krishnamurthy *et al.*, 2009), molecular dynamics (MD) simulations (Daily *et al.*, 2010; Whitford *et al.*, 2008), thermodynamics and unfolding (Rogne and Wolf-Watz, 2016; Schrank *et al.*, 2009), single molecule studies (Taylor *et al.*, 2018) (Lin *et al.*, 2013), hydrogen exchange mass spectrometry (Krishnamurthy *et al.*, 2009), various spectroscopies, mutagenesis (Bae and Phillips, 2006; Olsson and Wolf-Watz, 2010), evolution (Miller *et al.*, 2010) (Lange *et al.*, 1994), phylogeny (Saavedra *et al.*, 2018), and bioinformatics (Schulz *et al.*, 1986) and others have all been employed to gain insight regarding the connections between structure, dynamics, and function. Multiple timescales are involved and we seem to know only bits and pieces of relevant information.

Large scale motions seem to be dependent on more localized small-scale motions (Kerns *et al.*, 2015). Our results shed light on the range of motions that adenylate kinase undergoes during binding of substrates and their analogs. It has been appreciated for some time and more recently confirmed that AK's have lid domains of various sizes that open and close stochastically and the equilibrium shift to be more closed in response to binding of substrates and more recently studied (Daily *et al.*, 2012) (Kern *et al.*, 2005). We show here that, at least for the trimeric archaeal proteins, there is a room in the crystal lattice for substantial movement of these loops without disrupting the crystal packing. Because we have either 6 or 12 copies of each subunit in the asymmetric unit of our crystals, we have a rare opportunity to explore these ranges of structures. The multiple copies show slight variability in conformation but are smaller than the large shift in positions of the core, lid, and flap in going from an apo to a ligand bound state. First, we can overlay the multiple copies to observe the variation due to slightly different crystal environments, but we have also characterized the distributions that arise from the ensemble refinements.

The ensemble analysis shows that, even though the electron density map is very weak in areas of the protein, the constraints of the diffraction data combined with the molecular dynamics-like force field produce consistent patterns of inferred motions among the different subunits in the asymmetric unit. The superposition of the active site regions shows dancing guanidinium groups consistent with the hypothesis that changing hydrogen bonding patterns with the phosphate oxygen facilitate the phosphotransfer reaction. Our SVD analysis, via the left singular values, also shows fairly consistent transition pathways that can be visualized as “movies.” These movies provide hypotheses for describing the conformational landscape of lid, AMPbind, and side chain motions during catalysis. The observation that there is not one restricted hinge region but rather a collective reorganization of the moving parts is consistent with experimental data (mutagenesis, NMR, static crystal structures, etc.) and with computational studies that suggest highly distributed sets of bond angle rotations, but not completely random ones. In this work, we visualize a range of conformations likely to be relevant to the physiological motions and moves us closer to a complete description of the energy landscapes associated with the catalytic event in adenylate kinases.

The closest we can come to a parsimonious description of the generalized adenylate kinase catalytic cycle might proceed as follows. In the apo state, the lid and flap domains are undergoing openings and closings with some intermediates in somewhat higher energy states. The binding of the ligand, AMP selects for a more closed conformation of the flap, as does the binding of ATP for the lid domain. Mg++ is a required cofactor, bridging the beta and gamma phosphates of the ATP and helping to position them sterically and electronically for the phosphotransferase reaction. The actual electronic reconfigurations leading to the key transition state depend on the proper positioning of a critical guanidinium of a conserved arginine with the oxygen atoms of associated with the nucleotide phosphate oxygen atoms. Based on our observations and the molecular dynamics/quantum mechanics studies of Shibanuma *et al.* (2020), we support the hypothesis that a stochastic distribution of hydrogen bonded states between the key Arg residues and the phosphate oxygens are a part of the catalytic mechanism of action. They conclude “the structural flexibility of the protein, which allows recombination of hydrogen bonds within the catalytic scaffold, is essential for appropriately controlling the reaction” (Shibanuma *et al.*, 2020).

Product release can occur only with reopening of the lid and the flap, which exist on a broad, flat free energy landscape based on the mobility seen in our ensembles. The precise order of the large-scale binding events and the small-scale electronic positioning events is likely stochastic and variable from cycle to cycle and molecule to molecule. Our results show a range of conformations that are likely very similar in energy and, thus, members of the ensemble of states moving on the complex energy landscapes of the enzyme.

## Data Availability

The data that support the findings of this study are available from the corresponding authors upon reasonable request.
